# Leiomyosarcoma of the inferior vena cava level II involvement: curative resection and reconstruction of renal veins

**DOI:** 10.1186/1477-7819-10-120

**Published:** 2012-06-28

**Authors:** Quan Wang, Jing Jiang, Chao Wang, Guodong Lian, Mei-Shan Jin, Xueyuan Cao

**Affiliations:** 1 Department of General Surgery II, Jilin University First Hospital, Changchun, 130021, China; 2 Department of Pathology, Jilin University First Hospital, Changchun, 130021, China

**Keywords:** Leiomyosarcoma, Inferior vena cava, Renal veins, Reconstruction

## Abstract

Leiomyosarcoma of the inferior vena cava (IVCL) is a rare retroperitoneal tumor. We report two cases of level II (middle level, renal veins to hepatic veins) IVCL, who underwent en bloc resection with reconstruction of bilateral or left renal venous return using prosthetic grafts. In our cases, IVCL is documented to be occluded preoperatively, therefore, radical resection of tumor and/or right kidney was performed and the distal end of inferior vena cava was resected and without caval reconstruction. None of the patients developed edema or acute renal failure postoperatively. After surgical resection, adjuvant radiation therapy was administrated. The patients have been free of recurrence 2 years and 3 months, 9 months after surgery, respectively, indicating the complete surgical resection and radiotherapy contribute to the better survival. The reconstruction of inferior vena cava was not considered mandatory in level II IVCL, if the retroperitoneal venous collateral pathways have been established. In addition to the curative resection of IVCL, the renal vascular reconstruction minimized the risks of procedure-related acute renal failure, and was more physiologically preferable. This concept was reflected in the treatment of the two patients reported on.

## Background

Leiomyosarcoma of the inferior vena cava (IVCL) is a rare malignant tumor originating from the smooth muscle cells of the media with intraluminal or extraluminal growth. In the case of retroperitoneal occurrence, the middle portion of IVC is most frequently involved [1]. The origin of IVCL is further divided into three levels in relation to hepatic and renal veins: Level I, lower level (IVC below renal veins); Level II, middle level (renal veins to hepatic veins, most frequently affected); and Level III, upper level (entry of hepatic veins to right atrium) [2]. Patients with IVCL usually present with asymptomatic abdominal mass, therefore multiple diagnostic imaging techniques are used for the diagnosis, including Doppler ultrasonography, computed tomography (CT), and magnetic resonance imaging (MRI) [3].

Although the long-term survival is not that favorable (5-year survival 38%, 10-year survival 14%), the curative resection and possible adjuvant radiotherapy remain as the best treatment of choice [4]. The reconstructive regimen depends on the affected caval segment, the availability of retroperitoneal collateral circulation, and the invasion of adjacent structures, especially dictated by the involvement of unilateral or bilateral renal veins [5].

We herein report two cases of Level II IVCL with bilateral renal veins to explore the correlations between the radical resection of tumor, reconstruction of caval continuity, and the maximal preservation of renal functions.

## Case presentation

### Patient 1

**Figure 1:**
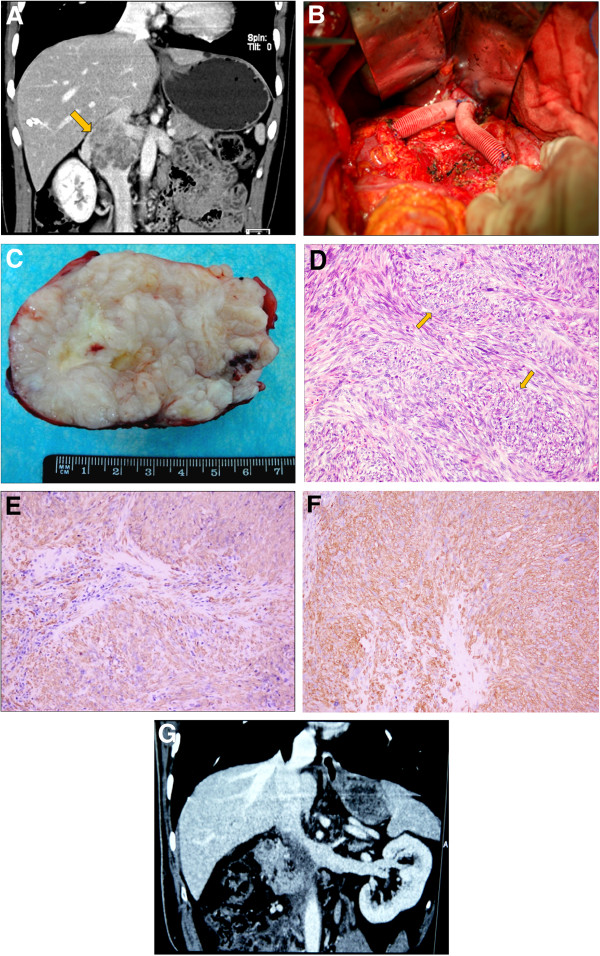
** Radiological, surgical, and pathological findings of leiomyosarcoma of the inferior vena cava.** (**A**) Contrast-enhanced CT scan revealed the leiomyosarcoma of the inferior vena cava (arrow) in case 1. (**B**) The resection of IVCL masses removed by the surgery in case 1. (**C**) Pathological view of the leiomyosarcoma: Moderately differentiated leiomyosarcoma with a fascicular growth pattern in case 1 (arrows). HE stain (×200). (**D**) The positive immunohistochemical staining for Caldesmon (×200) in case 1. (**E**) Immunohistochemical staining reveals the smooth muscle actin (SMA) expression in case 1; (×200). (**F**) The leiomyosarcoma of the inferior vena cava invading the right renal and left renal vein ostium in case 2. (**G**) Surgical view of the reconstruction of the bilateral renal veins with vascular prostheses; draining into proximal residual inferior vena cava in case 1.

A 53-year-old female patient, presented with vague abdominal pain for 6 months. She had a history of weight loss and had a palpable mass at the size 12 cm × 12 cm × 10 cm in her right upper quadrant, which was solid, less well-margined, poorly mobilized, and non-tender on palpation. CT scan showed a right retroperitoneal mass arising from inferior vena cava (Figure 1A). The intraluminal filling defects were detected indicating the tumor invaded into the inferior vena cava and bilateral renal veins. The renogram revealed normal renal function. No distention of abdominal subcutaneous vein was found. She was therefore diagnosed with retroperitoneal tumor, suspected leiomyosarcoma of the inferior vena cava, and received selective exploratory laparotomy. The tumor was explored to originate from the inferior vena cava, and infiltrated into bilateral renal veins. Thus, the inferior vena cava and bilateral renal veins were clamped and the tumor was subsequently removed. The distal end of inferior vena cava was ligated and excluded. The venous prosthetic grafts were inserted into the proximal end of inferior vena cava to redrain bilateral kidneys (Figure 1B). The postoperative pathological examination revealed retroperitoneal IVCL, moderate dysplasia with a margin free of tumor (Figure 1C,1D). The further immunohistochemical study confirmed the origination of 0by h-caldesmon(+), smooth muscle actin (+) (Figure 1E,1F) [6].

### Patient 2

A 66-year-old male patient was admitted to our department due to abdominal pain for 12 months. His physical examination was normal. CT scan showed the tumor invading the right renal and approaching left renal vein ostium (Figure 1G). The glomerular filtration rate examination and renogram revealed renal function was normal. He was therefore diagnosed with retroperitoneal tumor, suspected IVCL, and received selective exploratory laparotomy. The tumor originated from the inferior vena cava and was invading the right renal. Therefore, the IVC was clamped above and below the tumor followed by en bloc resection with the right kidney. Left renal vein reconstruction was lastly done using a graft. The postoperative pathological diagnosis of IVCL was confirmed using immunohistochemical examinations.

There were no postoperative complications and patients had an uneventful recovery. Two patients also received adjuvant radiotherapy at 2 Gray per fraction for 25 fractions to a total dose of 50 Gray. Anticoagulant therapy was continued for 3 months. Postoperative renal function confirmed by glomerular filtration rate assessment.

Two patients were followed up 27 months and 9 months, respectively, showing good performance status with normal renal function, absence of lower extremity edema, and no evidence of recurrent or metastatic disease on repeat imaging studies. Doppler ultrasonography also revealed no lower extremity venous stasis and thrombosis during the follow-up.

## Discussion

Curative resection remains the current treatment of choice for primary leiomyosarcoma of the IVC. However, the IVCL involving bilateral renal veins presents a surgical challenge. Palliative resections may temporarily improve symptoms but do not offer long-term survival. Aggressive radical resection is one choice for treatment [7]. Hines *et al*. reported improved survival with combined postoperative chemoradiation. However, the benefit of radiation, chemotherapy, or both for the treatment of IVC leiomyosarcoma is currently uncertain [8].

Surgical treatment should include complete resection of the malignant lesion with preservation of venous return. Following resection, there are several options available for the management of the Level II IVCL: complete ligation, or reconstruction with or without an arteriovenous fistula. Daylami *et al.* suggested that the resection of IVCL below hepatic veins should require no IVC reconstruction due to the abundant collateral circulation as the secondary lower extremity edema could be well tolerated [9].

In our cases, their tumor located at the IVC level II, which occupied 90% to 95% of the luminal diameter and invaded into bilateral renal veins in a mixed growth pattern. The patients complained of upper abdominal discomfort and/or had palpable mass but no lower extremity edema, indicating the likelihood of acceptable collateral circulation. Preoperative imaging also revealed a complete IVC occlusion, which is preferred to IVC ligation. The advantage is that the IVC ligation can reduce operative time, graft infection, high output cardiac failure from creation of a fistula, and the need for long-term anticoagulation. The disadvantage is that it results in lower extremity edema, which may be transitory [10].

The radical resection of the level II tumor requires consequent vascular reconstruction due to the frequent involvement of renal veins. The short right renal vein drains right ureteral vein, and its removal necessitating the sacrifice of right kidney. In contrast, the relatively long left renal vein is well collaterally circulated even in the case of complete IVC obliteration, allowing the preservation of left kidney. However, vascular prosthesis has been recommended to replace the affected renal vessels in aiming to minimize the postoperative occurrence of acute renal failure [11].

In case 1, due to the location of IVCL at the convergence of bilateral renal veins, the distal end of IVC was ligated followed by the complete removal of tumor. The bilateral renal veins were reconnected with the proximal end of IVC, maximizing the preservation of renal function as evidenced by follow-up renogram. The minimization of the interruption of renal outflow in the venous reconstruction was critical for the preservation of postoperative function [12]. In case 2, in the setting of concomitant right nephrectomy, ligation of left renal vein may lead to renal dysfunction and presents a management challenge to preserve as much renal function as possible. Left renal venous outflow reconstruction may avoid possible ischemic nephropathy and help preserve maximum renal function. It should especially be considered in presence of concomitant right nephrectomy.

## Conclusions

In conclusion, the complete surgical resection and radiotherapy offers the potential of better survival. In addition to the curative resection of IVCL, the renal vascular reconstruction minimized the risks of procedure-related acute renal failure, and was more physiologically preferable. This concept was reflected in the treatment of the two patients reported on.

## Consent

Written informed consent was obtained from the patient for publication of this case report and any accompanying images. A copy of the written consent is available for review from the Editor-in-Chief of this journal.

## Competing interest

The authors declare that they have no competing interests.

## Authors’ contributions

WQ and CXY wrote the main manuscript and performed the operation, JJ and JMS prepared the histological figures, WC and LGD provided the clinical history and clinical figures. All authors read and approved the final manuscript.
